# Impact of inflammatory status on intestinal iron absorption in older hospitalized patients

**DOI:** 10.1038/s41430-025-01604-2

**Published:** 2025-03-27

**Authors:** Baigang Wang, Rainer Wirth, Elena Bergmann, Lukas Funk, Chantal Giehl, Isabel Levermann, Gero Lueg, Tom Roloff, Maria Schnepper, Kiril Stoev, Rawi Zubi, Nina Rosa Neuendorff, Maryam Pourhassan

**Affiliations:** Department of Geriatric Medicine, Marien Hospital Herne, Ruhr-University Bochum, Hölkeskampring 40D, 44625 Herne, Germany

**Keywords:** Geriatrics, Nutrition

## Abstract

**Background and objective:**

Iron deficiency is prevalent among geriatric hospitalized patients, often coinciding with inflammation. This study aimed to determine a critical C-reactive protein (CRP) threshold for sufficient intestinal iron absorption using standardized tests.

**Subjects/Methods:**

This retrospective, cross-sectional study was conducted in a geriatric acute care unit. Serum iron and CRP levels were measured before breakfast and two- and four-hours after ingestion of two iron capsules. Intestinal iron absorption was calculated by subtracting baseline values from those obtained after the test, with an increase of 100 ug/dl indicating sufficient absorption. Patients were categorized into six CRP groups: ≤0.50, 0.51–2.50, 2.51–5.0, 5.1–7.50, 7.51–10.0, and ≥10.1 mg/dl.

**Results:**

The study included 59 participants (73% females, age range 71–99). Iron absorption was highest in groups with lower CRP levels ≤0.50 to 2.5 mg/dl) and declined significantly as CRP increased, particularly beyond 5 mg/dl. The most significant decline was noted in patients with CRP ≥ 10.1 mg/dl. A negative correlation between inflammation, as measured by CRP, and iron absorption was found. As CRP levels escalate, there is a significant reduction in the increase of serum iron levels after 2 h. A regression analysis showed that only elevated CRP levels significantly reduced serum iron increments post-iron supplementation (*P* = 0.004), while other factors such as age, sex, body mass index, frailty, weight loss, hemoglobin and nutritional status had no significant impact.

**Conclusion:**

A CRP level above 5 mg/dl is indicative of significantly impaired intestinal iron absorption in older patients, underscoring the critical influence of inflammation on iron metabolism.

## Introduction

Iron plays an essential role in cellular function and various physiological processes, such as haematopoesis and oxygen transport. Iron deficiency is a frequent disorder and regarded as one of the key factors of anemia, a worldwide public health problem [1]. Iron deficiency may occur on a continuum from latent iron deficiency without anemia to manifest iron deficiency with anemia and empty storages. An absolute iron deficiency is defined by a ferritin level <15 ng/ml in adults according to WHO-guidelines, whereas functional iron deficiency is defined by transferin saturation < 16%, independently from ferritin-levels [2, 3]. Functional iron deficiency is mostly induced by acute or chronic inflammation, leading to non-availability of iron stores even when storage iron is normal or increased [4]. Chronic diseases commonly associated with inflammation and functional iron deficiency include inflammatory bowel disease, chronic kidney disease, chronic liver disease, rheumatoid arthritis, and chronic heart failure [5]. Likewise, any acute disease with marked inflammation, such as infection, trauma, surgery may be associated with functional iron deficiency [6] and lead to inflammatory anaemia [7].

**Fig. 1:**
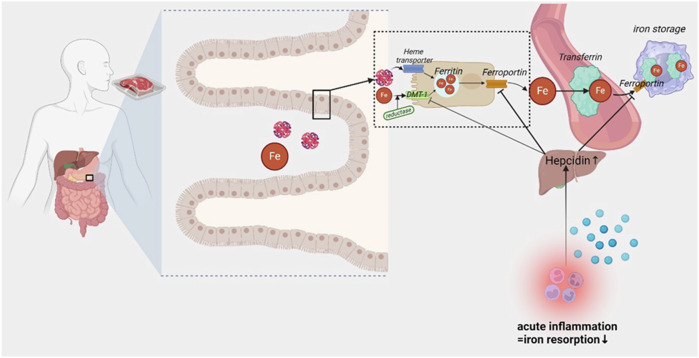
Intestinal iron absorption under physiological conditions and during inflammation. DMT-1 divalent metal transporter 1, Fe iron.

A primary regulatory mechanism for iron homeostasis involves the hepcidin-ferroportin axis [8] (Fig. 1). During inflammation, hepcidin—a negative regulator of ferroportin—is induced by circulating proinflammatory cytokines such as IL-6 [8, 9]. Consequently, iron becomes sequestered within the intracellular domains of hepatocytes and macrophages, as transmembrane iron transport via ferroportin is impaired. Furthermore, this pathway also disrupts intestinal iron absorption and systemic iron transport [10]. This mechanism serves an evolutionary role in host defense against bacterial infections, given that iron is crucial for bacterial growth. Therefore, iron supplementation during an infection might lead to adverse effects.

It has been demonstrated that bacterial growth is dependent on iron availability [11]. Therefore, iron supplementation is expected to support bacterial growth and increase the risk of infections [11], including enhancing mycobacterial growth [12]. Similarly, malaria is also strongly influenced by host iron [13, 14]. Recent research has also linked iron loading to atherosclerosis due to its role in the oxidation of biomolecules [15]. Conversely, iron deficiency has been associated with functional impairments [16, 17] and adverse health outcomes [18]. Evidence from prospective randomized controlled trials in patients with chronic heart failure has demonstrated that iron supplementation not only improves symptoms and functional capacity but also reduces mortality [19, 20]. Based on this evidence, the approach to iron supplementation—particularly its timing and method—is controversial, especially in settings involving intensive care and infectious diseases.

The severity of inflammation is associated with elevated levels of acute responsive proteins, such as C-reactive protein [21]. Many studies illustrated the relationship between the severity of inflammation and the effect of iron supplementation. Stoffel et al. demonstrated that in non-anemic women, iron-recycling macrophages are more sensitive than the enterocyte to high serum hepcidin during inflammation, induced by influenza/DPT vaccination [22]. A recent study showed that haemodialysis patients with CRP cutoff <0.1 mg/dl may benefit from oral iron supplementation and CRP > 0.1 mg/dl is associated with reduced efficiency [23].

In geriatric hospitalized patients, we face a high prevalence of iron deficiency, frequently coincident with inflammation. To test individual intestinal iron absorption, we occasionally use a standardized iron absorption test. To clarify which CRP cut-off value may be crucial for a sufficient intestinal absorption in oral iron supplementation, we analyzed the results of these iron absorption tests and their association with the degree of inflammation, measured by CRP, retrospectively.

## Subjects and methods

This cross-sectional study, conducted from October 2023 to February 2024, retrospectively analyzed the results of an intestinal iron absorption test performed on older hospitalized patients in the acute care ward of the university hospital, Marien Hospital Herne, Herne, Germany. Eligible patients were those who had a full iron assessment at admission, including serum iron, ferritin, transferrin, transferrin saturation, and C-reactive protein (CRP) measured on the day of the iron absorption test, with a transferrin saturation level below 16%. Patients were excluded if they had undergone iron replacement therapy within the previous three weeks or had conditions affecting iron absorption such as tetracycline therapy, gastric or small bowel resections, chronic inflammatory bowel disease, persistent diarrhea or vomiting, or known disorders of malabsorption or gastric emptying. All relevant data were extracted from the medical records of the patient.

### Laboratory measurements

Serum iron measurements were performed using the Cobas Pro (C503) analyzer by Roche, Mannheim, Germany, where the reference range for iron is established at 37–145 ug/dl. CRP measurements were performed on the same device with a reference value of <0.5 mg/dl. Patients were stratified into six groups based on their CRP levels at the time of the iron absorption test. These groups are defined as CRP ≤ 0.50, 0.51–2.50, 2.51–5.0, 5.1–7.50, 7.51–10.0, and ≥10.1 mg/dl.

### Iron absorption test

Serum iron and CRP levels were assessed early in the morning before breakfast to measure baseline values. Immediately following the initial blood collection, patients ingested two iron capsules, each containing 100 mg of iron(II) glycine sulfate (ferro sanol duodenal, UCB Pharma GmbH, Monheim, Germany) [24]. To minimize interference with iron absorption, patients were allowed to eat breakfast and take their usual medications thirty minutes post-ingestion, with the exception of calcium, magnesium, bicarbonate, and proton-pump inhibitors (PPIs). PPIs were also paused on the evening prior to the test. The effectiveness of iron absorption was subsequently evaluated by measuring serum iron levels at two and four hours after capsule ingestion. Absolute changes in iron levels were calculated by subtracting the baseline serum iron levels from those measured after 2 and 4 h. Additionally, percentage changes in serum iron after 2 and 4 h were calculated relative to the initial serum iron values. An increase in serum iron of 100 ug/dl (equivalent to 18 μmol/L) from baseline was considered indicative of positive iron absorption [24].

### Statistical analysis

The statistical analysis was performed with SPSS statistical software (SPSS Statistics for Windows, IBM Corp, Version 29.0, Armonk, NY, USA). Sample size was determined with consideration for detecting significant differences in iron absorption across CRP groups, balanced against the constraints of the retrospective study design. Ten patients were selected for each group, with one group containing 9 patients, totaling 59 participants, to ensure a robust analysis across CRP categories. While the lack of a formal power analysis is a limitation due to the study’s retrospective nature, our approach was constrained by the data available. As this study served as a pilot project without prior data to predict differences between the groups, we utilized all available data to optimize our analytical approach.

Continuous variables are reported with means and standard deviations (SD) for normally distributed variables and median values with interquartile ranges (IQR) for non-normally distributed data. Categorical variables are expressed as absolute numbers and relative frequencies (%). Differences in serum iron levels from baseline to 2 and 4 h post iron absorption test across each CRP category were evaluated using repeated measures ANOVA, with Tukey’s post-hoc tests applied to identify specific group differences. Furthermore, one-way ANOVA was used to compare both absolute and percentage changes in serum iron levels after 2 and 4 h post iron absorption test across different CRP groups. Additionally, multiple regression analysis was used to examine the impact of various risk factors, such as age, gender, BMI, frailty, nutritional status, weight loss, Hb, inflammation and serum ferritin (as independent variables), on changes in serum iron 2 h post iron absorption test (as the dependent variable). Statistical significance was set at *p* < 0.05.

## Results

Baseline characteristics of study participants are presented in Table 1. The total study population consisted of 59 participants, with an age range of 71–99 years (73% females). According to MNA-SF, 42% of patients were classified as malnourished, another 42% were at risk of malnutrition, and 16% had a normal nutritional status. Hb levels show no sex differences between both males and females (*P* = 0.973); however, the prevalence of anemia is high, affecting 69% (*n* = 11) of males and 63% (*n* = 27) of females within the study group. In addition, 68% (*n* = 38) of the patients were identified as frail based on the clinical frailty scale. There were no significant differences between CRP levels on admission and at the time of iron absorption test (*P* = 0.437).

**Table 1:** Characteristics of the study population.

	Total population (*n* = 59)
Gender	
Female (*n*; %)	43 (73)
Male (*n*; %)	16 (27)
Age (y)	82.7 ± 7.8
Height (m)	1.65 ± 0.09
Actual body weight (kg)	71.2 ± 20.2
BMI (kg/m^2^)	25.7 ± 6.1
Hb (g/dl)	
Female	11.7 ± 3.5
Male	11.7 ± 2.0
Ferritin (ng/ml)	271.4 ± 282.0
Clinical Frailty Scale	6 (5-6)
MNA-SF, Median (IQR)	8 (6-11)
Malnourished (*n*; %)	24 (42)
At risk of malnutrition (*n*; %)	24 (42)
Normal nutritional status (*n*; %)	9 (16)
CRP (mg/dl)	
On admission	5.0 ± 4.1
At the time of iron absorption test	5.7 ± 5.0

Values are given as mean ± SD, number (%) or median (IQR, interquartile range).

*ASBMI* body mass index, *Hb* hemoglobin, *MNA-SF* Mini Nutritional Assessment Short Form, *CRP* C-reactive protein.

Table 2 shows a detailed analysis of iron absorption patterns across different levels of CRP, with measurements taken before, 2 h after, and 4 h after an iron absorption test. The results indicate that average iron absorption is high in the groups of patients with lower CRP levels (≤0.50 mg/dl, 0.51–2.50 mg/dl, and 2.51–5.0 mg/dl), in which iron concentrations significantly increase from baseline through 2 h and maintain at 4 h. However, a notable decline in iron absorption efficiency is observed as CRP levels rise to 5.1–7.50 mg/dl. This decline becomes more evident in the 7.51–10.0 mg/dl CRP group, where the variability in response suggests less absorption patterns. Notably, within this group, 4 patients demonstrate higher iron absorption after 2 and 4 h, indicating that despite elevated levels of inflammation, some individuals may still manage to absorb iron effectively. For individuals with CRP levels exceeding 10.1 mg/dl, the absorption is markedly compromised, showing the pronounced adverse effect of high-level inflammation on iron metabolism.

**Table 2:** Detailed analysis of iron absorption patterns across different levels of CRP at the time of iron absorption test.

	Iron levels			
CRP groups (mg/dl)	Before test ug/dl	After 2 h ug/dl	After 4 h ug/dl	*P* value between group
CRP ≤ 0.50	60.5 ± 17.6	166.4 ± 88.5	161.9 ± 91.0	0.002
CRP 0.51–2.50	46.2 ± 17.0	159.0 ± 97.7	154.2 ± 85.1	0.003
CRP 2.51–5.0	42.3 ± 15.6	109.9 ± 54.6	106.2 ± 72.6	0.002
CRP 5.1–7.50	33.2 ± 13.6	57.4 ± 27.8	62.7 ± 31.6	0.002
CRP 7.51–10.0	37.9 ± 24.5	86.5 ± 43.5	108.4 ± 99.6	0.083
CRP ≥ 10.1	20.3 ± 11.5	38.3 ± 45.5	37.7 ± 49.2	0.970

Differences in serum iron levels from baseline to 2 and 4 h post iron absorption test across each CRP category were evaluated using repeated measures ANOVA, with Tukey’s post-hoc.

*CRP* C-reactive protein (%).

**Fig. 2:**
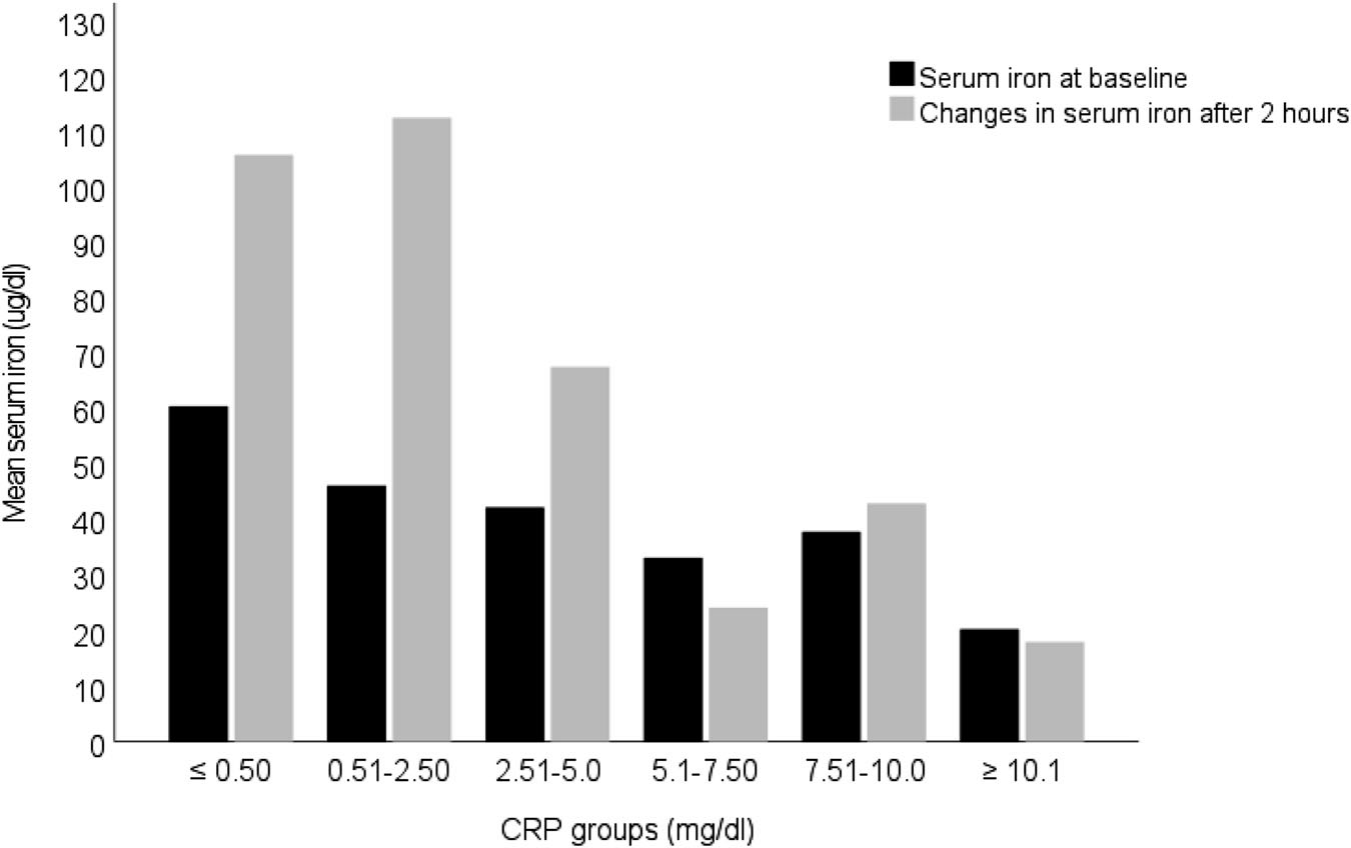
Baseline serum iron levels and changes after 2 h across different CRP groups. CRP C-reactive protein.

Figure 2 shows that individuals with lower CRP levels (≤0.50 and 0.51–2.50 mg/dl) have higher baseline and post-test iron levels compared to those in higher CRP categories. Notably, in groups with CRP levels above 5 mg/dl, both, baseline and change in iron levels, are significantly lower, suggesting the negative impact of inflammation on iron absorption and overall iron status.

**Fig. 3:**
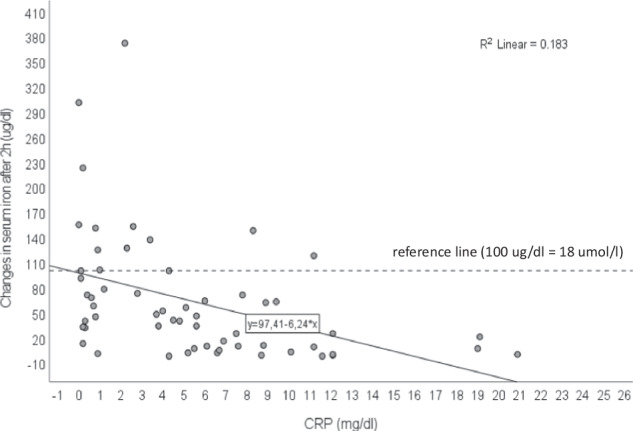
Correlation between CRP levels at the time of iron absorption test and changes in serum iron after 2 h. CRP C-reactive protein.

Absolute and percentage changes in serum iron levels after 2 and 4 h, categorized by different CRP concentrations, are presented in Table 3. The data demonstrate a statistically significant decline in the absolute changes in serum iron as CRP levels increase, with notable decreases observed both at 2 h (*P* = 0.006) and 4 h (*P* = 0.049) after the iron absorption test. In contrast, the percentage changes in serum iron, although following a similar trend, do not exhibit statistically significant differences across CRP groups (*P* = 0.170 at 2 h and *P* = 0.188 at 4 h). The observed pattern in Fig. 3 confirms these findings, highlighting a clear, negative correlation between inflammation and the efficiency of iron absorption over time. As CRP levels escalate, there is a significant reduction in the increase of serum iron levels after 2 h.

**Table 3:** Absolute and percentage changes in serum iron after 2 and 4 h across the CRP groups.

	Changes in serum iron after 2 h		Changes in serum iron after 4 h	
CRP groups (mg/dl)	ug/dl	%	ug/dl	%
CRP ≤ 0.50	105.9 ± 93.9	206.9 ± 216.7	101.4 ± 95.4	194.8 ± 216.9
CRP 0.51–2.50	112.6 ± 101.3	316.6 ± 428.8	108.0 ± 89.9	306.0 ± 388.2
CRP 2.51–5.0	67.6 ± 48.6	173.5 ± 144.8	63.9 ± 62.8	145.3 ± 133.6
CRP 5.1–7.50	24.2 ± 23.8	81.2 ± 76.3	29.5 ± 29.0	95.5 ± 90.4
CRP 7.51–10.0	48.6 ± 48.8	205.2 ± 230.1	70.5 ± 109.3	314.7 ± 474.8
CRP ≥ 10.1	18.0 ± 36.3	62.7 ± 92.2	17.4 ± 40.3	54.8 ± 95.7
*P* value (between group)	0.006	0.170	0.049	0.188

*CRP* C-reactive protein.

To test the independent effect of various risk factors such as age, gender, BMI, frailty, nutritional status, weight loss, Hb, and inflammation and serum ferritin on changes in serum iron 2 h post iron absorption test, we performed a regression analysis (Table 4). Among all factors, inflammation (CRP, *P* = 0.022) and serum ferritin (*P* = 0.033) significantly impacted the increase of serum iron levels after oral iron ingestion. Increased CRP levels were associated with a significantly lower increase in serum iron, while variations in ferritin levels also showed a notable impact. None of the other variables showed a statistically significant effect on serum iron changes after 2 h.

**Table 4:** Regression analysis of risk factors associated with changes in serum iron 2 h after an iron absorption test.

	Changes in serum iron after 2 h ug/dl					
				95% CI for Exp(B)		
	B	Std. Error	Exp(B)	Lower	Upper	*P* value
Age (year)	−0.946	1.385	−0.090	−3.746	1.853	0.498
Gender (female/male)	29.538	22.430	0.171	−15.795	74.870	0.195
BMI (kg/m^2^)	0.504	1.752	0.042	−3.037	4.044	0.775
Clinical Frailty Scale	−6.166	8.990	−0.094	−24.335	12.003	0.497
Total MNA-SF	4.886	4.570	0.189	−4.350	14.122	0.291
Previous weight loss-MNA-SF	0.952	14.634	0.011	−28.623	30.528	0.948
Hb (g/dl)	−5.482	2.895	−0.239	−11.332	0.368	0.065
CRP, at the iron tolerance test (mg/dl)	−4.959	2.074	−0.341	−9.150	−0.768	0.022
Ferritin (ng/ml)	−0.075	0.034	−0.293	−0.143	−0.006	0.033

*BMI* body mass index, *MNA-SF* Mini Nutritional Assessment Short Form, *d-iron* changes in serum iron after 2 h.

## Discussion

The inhibitory effects of inflammation on iron absorption and metabolism are well-documented. However, the precise degree of inflammation and the levels of inflammatory markers that define a threshold for impaired intestinal iron absorption remains undefined. In clinical practice, an iron-deficient patient presenting with concurrent inflammation is common. While intravenous iron therapy is an alternative, it is associated with a significant risk of allergic reactions and elevated healthcare costs compared to oral iron supplementation. Therefore, establishing a definitive CRP threshold could significantly enhance clinical decision-making.

This retrospective study sought to identify a CRP cutoff that correlates with impaired intestinal iron absorption. We observed markedly reduced iron absorption in patients with CRP levels above 10 mg/dl and noted a significant reduction of intestinal iron absorption starting from CRP levels of 5.1–7.50 mg/dl. These findings suggest a CRP threshold of 5 mg/dl for identifying impaired intestinal iron absorption. Notably, the group with CRP levels between 7.5 and 10 mg/dl exhibited adequate, albeit delayed, iron absorption, likely influenced by outliers and the limited sample size within these groups. Furthermore, our study did not distinguish between acute and chronic inflammation or analyze the dynamics of CRP changes. Given that impaired iron absorption plays a protective role during bacterial infections, the underlying causes of CRP elevation could significantly impact the extent of iron absorption impairment. Further, we considered the influence of low-grade inflammation related to adiposity on iron absorption, which may be mediated by increased hepcidin expression [25]. Prior research indicates that inflammation associated with obesity and overweight can reduce iron absorption and diminish the effectiveness of iron-fortified foods [26]. However, in our study, BMI did not significantly impact iron absorption, as determined by regression analysis. Future research should aim to clarify the impact of the underlying causes of CRP elevation and its dynamics on iron absorption, preferably in larger, prospective studies.

Even with adequate iron levels after supplementation, the recovery from iron-deficiency anemia is influenced by several factors, including bone marrow reserve, concurrent myelosuppressive medications, and the severity of any acute illness, particularly in older patients. Consequently, our study concentrated solely on the dynamics of iron absorption and omitting further analysis of hemoglobin increments in patients with clinically evident anemia. Nevertheless, it has been observed that patients with CRP levels above 0.4 mg/dl experience delayed increases in hemoglobin compared to those with lower CRP [27]. Although this threshold significantly deviates from ours, it emphasizes the influence of elevated CRP on impaired iron absorption. Interestingly, iron absorption efficiency is also dependent on iron storage levels, improving with ferritin levels below 30 ng/ml and worsening above 100 ng/ml [28]. In the context of our findings, both CRP and serum ferritin emerged as significant determinants of iron absorption efficiency. While CRP is a well-known acute-phase reactant indicating inflammation, ferritin serves as an indicator of iron stores and is influenced by both iron status and inflammatory conditions [29]. Our analysis further highlights the complex interplay between inflammation (as indicated by CRP) and iron stores (as reflected by ferritin levels) in regulating iron absorption. Notably, the regression results demonstrate that the association of CRP with impaired iron absorption remains robust and independent of iron status (severity of depletion). This independence underscores the critical role of acute inflammatory responses in influencing iron absorption.

Despite certain limitations as discussed above, a key strength of our investigation lies in its specific focus on direct measures of intestinal iron absorption without resorting to surrogate markers like haemoglobin increments, thus minimizing potential confounding factors. Consequently, we recommend caution in using oral iron supplements for patients with a CRP level above 5 mg/dl, particularly in older patients with multiple comorbidities. Maintaining adequate iron stores is crucial in such populations, e.g., for its role in myoglobin and therefore its association with muscle strength and function in older hospitalized individuals [17]. Furthermore, subclinical iron deficiency may contribute to conditions like restless-legs syndrome [30], which could be erroneously diagnosed as delirium in critically ill older adults patients. Recent studies have demonstrated that intravenous iron supplementation can significantly improve common symptoms such as fatigue in older patients [31]. Collectively, these findings underscore the vital roles of iron in the human body and highlight the need for careful management of iron supplementation in older hospitalized patients.

## Conclusion

Our study conclusively identifies a CRP threshold of greater than 5 mg/dl as indicative of markedly impaired intestinal iron absorption in older hospitalized patients, highlighting the significant impact of inflammation on iron metabolism.

## Data Availability

The datasets analyzed during the current study are available from the corresponding author on reasonable request.
